# The effect of different classification of hospitals on medical expenditure from perspective of classification of hospitals framework: evidence from China

**DOI:** 10.1186/s12962-020-00229-5

**Published:** 2020-09-16

**Authors:** Lele Li, Tiantian Du, Yanping Hu

**Affiliations:** 1 School of Public Policy and Management, Tsinghua University, 1 Tsinghua Yard, Haidian District, Beijing, China; 2 Institute for Hospital Management, Tsinghua University, 1 Tsinghua, Nanshan District, Shenzhen City, Guangdong Province China; 3 Department of Medical Engineering, China-Japan Friendship Hospital, 2 Yinghua Yuan, Chaoyang District, Beijing, China

**Keywords:** Classification of hospitals, Medical expenditure, Medical outcome; multivariate regression model, Tiered delivery system

## Abstract

**Background:**

Different classification of hospitals (COH) have an important impact on medical expenditures in China. The objective of this study is to examine the impact of COH on medical expenditures with the hope of providing insights into appropriate care and resource allocation.

**Methods:**

From the perspective of COH framework, using the Urban Employee Basic Medical Insurance (UEBMI) data of Chengdu City from 2011 to 2015, with sample size of 488,623 hospitalized patients, our study empirically analyzed the effect of COH on medical expenditure by multivariate regression modeling.

**Results:**

The average medical expenditure was 5468.86 Yuan (CNY), the average expenditure of drug, diagnostic testing, medical consumables, nursing care, bed, surgery and blood expenditures were 1980.06 Yuan (CNY), 1536.27 Yuan (CNY), 500.01 Yuan (CNY), 166.23 Yuan (CNY), 221.98 Yuan (CNY), 983.18 Yuan (CNY) and 1733.21 Yuan (CNY) respectively. Patients included in the analysis were mainly elderly, with an average age of 86.65 years old. Female and male gender were split evenly. The influence of COH on total medical expenditures was significantly negative (*p* < 0.001). The reimbursement ratio of UEBMI had a significantly positive (*p* < 0.001) effect on various types of medical expenditures, indicating that the higher the reimbursement ratio was, the higher the medical expenditures would be.

**Conclusions:**

COH influenced medical expenditures significantly. In consideration of reducing medical expenditures, the government should not only start from the supply side of healthcare services, but also focus on addressing the demand side.

## Background

Controlling the unreasonable growth of medical expenditures has become an important goal and critical issue in healthcare reform worldwide. In China, medical expenditures continue to mount. According to the report *The Forecast and Governance of China's Medical Expenditure Growth*, medical expenditure per capita rose from 30.2 Yuan (CNY) in 1991 to 1047.9 Yuan (CNY) in 2013, with an average annual growth rate of 17.49%, 2% higher than the average 15.12% per capita annual crude growth of the gross domestic product (GDP) during the same timeframe. Medical expenditures are paid by Governement Medical Insurance in China, which cover more than 95% of the Chinese population. Soaring medical expenditures will not only increase the financial burden, reduce the affordability of medical services and the sustainability of medical insurance, but also hinder the improvement of population health. Therefore, it is an important research subject for the Healthy China strategy [1] to analyze factors causing the increase of medical expenditure and; to put forth suggested solutions to control them.

In November 1989, the former Ministry of Health (Now Called the National Health Commission) enacted *The measures for the administration of the hospital grade (trial draft)* [2], which implemented COH in China in order to provide clarity and structure. COH divided hospitals into Grade I, II and III hospitals according to their functions and roles. Grade I hospitals include community health centers and township health centers that directly provide prevention, medical care and rehabilitation services to residents. Grade II hospitals are secondary hospitals that provide comprehensive medical services to a region, undertake some teaching and scientific research tasks. Grade III hospitals are tertiary hospitals that provide high-level specialized medical services and undertake advanced teaching and scientific research tasks. Further, these three grades are subdivided into 3 subsidiary grades, including A, B and C based on the hospital’s scale, service provision, medical technology and equipment, medical research and so on.

COH has played a positive role in establishing an efficient healthcare administration system, and in strengthening the three-tier prevention healthcare network [2], providing convenient and suitable medical services. But it has created a problem of uneven distribution of medical resources under COH. There has been a significant difference in government’s investment, medical technology, and social reputation (people’s trust in hospitals) across COH. In China, the hospital level has various influences on the allocation of medical resources, which can not only affect the government subsidies for hospitals, but also the hospital development and health seeking behavior of patients. It is well known that there is a large gap among COH related to medical expenditures [2, 3]. According to the classification standard of hospitals in China, big hospitals mainly refer to hospitals which provide advanced medical services, and undertake teaching and scientific research [3].

Therefore, it is the intention of this analysis to examine the effect of COH on medical expenditures; as there have been no studies exploring this effect; especially in China. For the purposes of this paper, hospitals were divided into three levels, including Grade II Level A hospital, Grade II Level B hospital, and Grade II Level C hospital. Per the classification standards of hospitals in China, big hospitals in our study were Grade II Level A hospitals, which were the largest scale hospitals, providing the best service, the most advanced medical technology and equipment, and the best medical research within all the Grade II hospitals. This was due to data limitations of Grade III hospitals.

## Methods

### Data source and variable descriptions

The data was derived from UEBMI of Chengdu City from 2011 to 2015. The sample size was 1,035,556 hospitalized patients. We studied the influence of COH on medical expenditures based on the premise of a full patient recovery, including patients discharged from the hospital. Therefore, the 488,667 patients with recovery results in the sample were selected for analysis, with a sample size of 488,623 after outliers and missing values including death,transfer,and other situations were deleted.

The dependent variable used was medical expenditures, which consisted of drug, diagnostic testing, medical consumables, nursing care, bed, blood and surgery expenditures. The independent variable was hospital level, which was designated according to the classification standard of hospitals in China. Price factors and patients characteristic factors were defined as control variables. Control variables refer to all the varibles that affect the results of dependent varibles except for independent variables. Only by controlling all the variables other than independent variables that can cause the dependent variable to change, can the causality be clarified between COH and HE in the regression analysis.The price factor included the reimbursement ratio (defined below) and the deductible was defined as a potential factor that affected the medical expenditure.

The reimbursement ratio is the proportion of medical expenditures covered by medical insurance of the total medical expenditure. The deductible (portion patient’s self-pay) was closely related to the hospital level and thus in different levels of hospitals, the deductible was different. Chengdu City had implemented the medical insurance payment methods reform and used diagnosis related groups (DRGs). DRGs are based on resource consumption and disease severity by ICD-10 diagnosis codes. In order to control for disease severity, we divided the disease into three types, including chronic disease, critical disease and common disease. So, disease type reflected the the disease severity and patients characteristic factors included age, gender, length of stay, and disease type.

### Model structure

In order to evaluate the effect of COH on medical expenditures, our study set up the model as follows (Eq. 1).

1 $$Expenditure_{i} = \beta_{0} + \beta_{1} rank_{i} + \beta_{2} disease_{i} + \beta_{3} X_{i} + \varepsilon_{i}$$

In order to evaluate whether the effect of COH on medical expenditures was affected by disease type, the regression model was set as follows (Eq. 2).

2 $$Expenditure_{i} = \beta_{0} + \beta_{1} rank_{i} + \beta_{2} disease_{i} + \beta_{3} rank \times disease_{i} + \beta_{4} X_{i} + \varepsilon_{i}$$

In order to evaluate whether the effect of COH on medical expenditures was affected by the reimbursement ratio, the model was set as follows (Eq. 3):

3 $$Expenditure_{i} = \beta_{0} + \beta_{1} rank_{i} + \beta_{2} disease_{i} + \beta_{3} rank \times Reimbursement_{i} + \beta_{4} X_{i} + \varepsilon_{i}$$

The following definitions applied:${\beta }_{0}-{\beta }_{4}$ denotes model coefficient, ${Expenditure}_{i}$ denotes the medical expenditure of patients (including total medical expenditures, made up of drug, diagnostic testing, medical consumables, nursing care, bed, blood and surgery expenditures).${rank}_{\mathrm{i}}$ denotes the hospital level, including Grade II Level A hospital, Grade II Level B hospital, and Grade II Level C hospital.. $rank\times {disease}_{i}$ denotes the interaction item between hospital levels and disease type. $rank\times {Reimbursement}_{i}$ denotes interaction item between hospital levels and the reimbursement ratio. ${\mathrm{X}}_{\mathrm{i}}$ denotes a group of observable control variables, including the reimbursement ratio, the deductible, age, gender, length of stay, and disease type and ${\upvarepsilon }_{\mathrm{i}}$ denotes the error term.

In order to test whether the effect of hospital level on medical expenditure was affected by disease type and reimbursement ratio, this study utilitzed multivariate regression analysis by adding the interaction item between hospital level and disease type as well as interaction item between hospital level and reimbursement ratio.

## Results

### Descriptive statistics

As seen in Table 1, the average medical expenditure was 5468.86 Yuan (CNY), the average expenditures of drug, diagnostic testing, medical consumables, nursing care, bed, surgery and blood expenditures were 1980.06 Yuan (CNY), 1536.27 Yuan (CNY), 500.01 Yuan (CNY), 166.23 Yuan (CNY), 221.98 Yuan (CNY), 983.18 Yuan (CNY) and 1,733.21 Yuan (CNY) respectively. The number of Grade II Level A hospitals accounted for the highest proportion of hospitals. The patients were mainly elderly, with an average age of 86.65 years old. The average length of stay was 9.42 days. Female and male were split evenly. In terms of the reimbursement ratio, the actual reimbursement ratio of Grade II Level A hospitals was higher than that of Grade II Level B hospitals and Grade I hospitals. Surprisingly, the reimbursement ratio of Grade II Level B was the lowest among all of hospitals. In terms of deductible, the higher the hospital level, the higher the deductible. Disease types included chronic disease, critical disease and common disease refecting the disease severity. Chronic disease and critical disease comprised 5% and common disease 90% of disease types.

**Table 1:** The results of descriptive statistics

Variable type	Variable name	Variable definition	Mean		SD	Minimum	Maximum
Dependent variable							
Medical expenditure	Total medical expenditure	Average total medical expenditure	5468.86	5820.13		0.35	559,328.4
	Drug expenditure	Average drug expenditure	1980.06	2366.27		0.01	157,170.3
	Medical consumables expenditure	Average medical consumables expenditure	500.01	1806.15		0.05	95,490.39
	Diagnostic testing expenditure	Average diagnostic testing expenditure	1536.27	1546.74		2	156,887
	Nursing care expenditure	Average nursing care expenditure	166.23	250.38		0.34	73,077
	Bed expenditure	Average bed expenditure	221.98	222.42		2	47,880
	Blood expenditure	Average blood expenditure	1733.21	1386.78		70	49,813.23
	Surgery expenditure	Average surgery expenditure	983.18	988.32		0.4	25,591.9
Inpendent variables							
Hospital level	Grade II Level A hospital	Grade II Level A hospital = 1, otherwise = 0	0.83	0.37		0	1
	Grade II Level B hospital	Grade II Level B hospital = 1, otherwise = 0	0.12	0.33		0	1
	Grade II Level C hospital	Grade II Level C hospital = 1, otherwise = 0	0.03	0.16		0	1
Control variables							
Reimbursement ratio	Grade II Level A hospital	Average reimbursement ratio Grade II Level A hospital	83.95%	0.14		0.15%	100%
	Grade II Level B hospital	Average reimbursement ratio Grade II Level B hospital	76.70%	0.15		0.63%	100%
	Grade II Level C hospital	Average reimbursement ratio Grade II Level C hospital	82.90%	0.15		2.35%	100%
Deductible	Grade II Level A hospital	Average deductible of Grade II Level A hospital	271.90	95.19		0	400
	Grade II Level B hospital	Average deductible of Grade II Level B hospital	241.65	83.22		0	400
	Grade II Level C hospital	Average deductible of Grade II Level C hospital	184.50	93.08		0	400
Patients characteristic	Gender	female = 1, others = 0	0.52	0.50		0	1
	Age	Age of patient	85.65	23.92		0	133
	Length of stay	Actual length of stay	9.42	44.67		1	23,070
Disease type	Chronic disease	Chronic disease = 1, otherwise = 0	0.05	0.21		0	1
	Critical disease	Critical disease = 1, otherwise = 0	0.05	0.21		0	1
	Common disease	Common disease = 1, otherwise = 0	0.90	0.29		0	1

Reimbursement ratio = Actual reimbursable expenses/Eligible reimbursable expenses

### Empirical results

In order to evaluate the effect of COH on medical expenditures, our study used UEBMI data from Chengdu City by multivariate regression model. As shown in Table 2, in terms of the hospital characteristic factor, hospital level had a significant influence on medical expenditures. Furthermore, the influence of hospital level on total medical expenditure was significantly negative (*p* < 0.001). The higher the hospital level was, the lower the medical expenditures were. Hospital level was significantly positive for surgery expenditure (*p* < 0.001), i.e. the higher the hospital level, the higher the surgery expenditure. Additionally, high-level hospitals performed a large number of surgeries for complicated or emergent conditions. So, the higher the hospital level, the higher the surgery expenditure.

**Table 2:** The effect of hospital level on medical expenditure

Variables	Medical expenditure							
	Total medical expenditure	Drug expenditure	Diagnostic testing expenditure	Medical consumables expenditure	Nursing care expenditure	Bed expenditure	Surgery expenditure	Blood expenditure
Hospital level	− 0.2311*** (− 160.86)	− 0.1884*** (− 76.30)	− 0.4240*** (− 178.31)	− 0.3235*** (− 71.23)	− 0.0197*** (− 12.46)	− 0.0293*** (− 19.04)	0.2114*** (15.68)	− 0.1186*** (− 5.17)
Reimbursement ratio	2.8313*** (476.43)	3.4665*** (337.93)	2.8306*** (287.83)	2.2364*** (120.75)	1.1725*** (179.05)	1.3738*** (214.24)	1.8895*** (15.68)	0.5061*** (8.48)
Deductible	− 0.0005*** (− 70.09)	− 0.0012*** (− 90.65)	0.0002*** (17.58)	− 0.0019*** (− 84.40)	− 0.0007*** (− 77.86)	− 0.0002*** (− 29.67)	− 0.0007*** (− 10.28)	− 0.0009*** (− 10.34)
Gender	− 0.0217*** (− 16.09)	− 0.0579*** (− 24.92)	0.0084*** (3.81)	− 0.0852*** (− 20.33)	0.0052*** (3.50)	0.0088*** (6.05)	0.1260*** (11.71)	− 0.0779*** (− 5.89)
Age	0.0034*** (107.11)	0.0043*** (79.10)	0.0106*** (202.17)	0.0009*** (10.06)	− 0.0008*** (− 23.31)	− 0.0012*** (− 36.24)	− 0.0010*** (− 3.82)	− 0.0018*** (− 4.80)
Length of stay	0.0375*** (390.27)	0.0442*** (266.54)	0.0146*** (92.71)	0.0188*** (62.68)	0.0565*** (527.47)	0.0581*** (558.19)	0.0130*** (18.61)	0.0008 (1.49)
Disease type	− 0.0092*** (− 6.18)	− 0.0655*** (− 25.71)	− 0.1762 (− 72.91)	0.2513*** (54.76)	− 0.0079*** (− 4.87)	− 0.0157*** (− 9.85)	1.0800*** (55.24)	0.0726*** (3.84)
Time	Controlled	Controlled	Controlled	Controlled	Controlled	Controlled	Controlled	Controlled
Constant term	− 5.8625*** (− 17.53)	− 8.6725*** (− 15.10)	− 1.4927*** (− 2.74)	− 6.0105*** (− 5.83)	3.2771*** (6.34)	3.3938*** (6.73)	− 6.9009*** (− 3.66)	7.0667*** (11.32)
R^2^	0.6395	0.4556	0.4525	0.0941	0.5034	0.5337	0.0461	0.0265
Sample size	488,623	486,558	483,777	483,797	488,166	486,079	117,194	8885

***Significant at 1%,**Significant at 5%, *Significant at 10%, t-statistics in parentheses. The medical expenditure in the model are all calculated on a logarithmic basis

In terms of the medical price factor, the reimbursement ratio had a significantly positive effect on various types of medical expenditures (*p* < 0.001), indicating that the higher the reimbursement ratio was, the higher the medical expenditure was. The effect of the deductible on various types of medical expenditures was significantly negative (*p* < 0.001) except for diagnostic testing expenditures, indicating that the higher the deductible was, the lower the medical expenditure. Lastly the effect of the deductible on diagnostic testing expenditures was significantly positive (*p* < 0.001).

In terms of the individual characteristic factors, the effect of gender and age on medical expenditures was inconsistent. Males spent more than females in terms of total medical expenditure, drug, medical consumables and blood expenditure. However, males spent less than females on diagnostic testing, nursing care, bed and surgery expenditure. The older the age, the higher the total medical expenditure for drugs, diagnostic testing and medical consumables (*p* < 0.01). Conversely the older the age, the lower the nursing care, bed, surgery and blood expenditures. The increase of medical expenditures had a near-death effect. That is, elderly approaching the end of life have a higher increase of medical expenditure [46]. The length of stay had a significantly positive effect on medical expenditure (*p* < 0.01). The impact of disease types including chronic, critical and common disease on medical expenditure were different. The more serious the disease type, the higher the total medical expenditures of drug, diagnostic testing, nursing care and bed. Interestingly, the more serious the disease type, the lower the medical consumables, surgery and blood expenditure.

The empirical results are shown in Tables 3, 4 and 5 for the effect of hospital level on medical expenditure per hospital classification. The effect of Grade II Level A hospitals on the total medical expenditure and surgery expenditure are significantly negative (*p* < 0.001), while the effect on drug, diagnostic testing and medical consumables expenditure are significantly positive (*p* < 0.001). The effect of Grade II Level B hospitals on total medical expenditure, drug and diagnostic testing expenditure were significantly negative (*p* < 0.001); while the effect on medical consumables, nursing care, bed and surgery expenditure were significantly positive (*p* < 0.001). The effect of Grade II Level C hospitals on total medical expenditures of drug, diagnostic testing and blood expenditure were significantly negative (*p* < 0.001); while the effect on medical consumables, nursing care, bed and surgery expenditure were significantly positive (*p* < 0.001). After distinguishing for COH, hospitals of different levels had different influences on medical expenditure. The effect of Grade II Level A hospitals, Grade II Level B hospitals, Grade II Level C hospitals per below (Tables 4, 5, 6) on the total medical expenditure were significantly negative (*p* < 0.001). On the whole, the higher the hospital level, the lower the total medical expenditure. The effect of the reimbursement ratio, deductible, gender, age, length of stay and disease type on medical expenditure were consistent with the previous analysis.

**Table 3:** The effect of Grade II Level A hospitals on medical expenditure

Variables	Medical expenditure							
	Total medical expenditure	Drug expenditure	Diagnostic testing expenditure	Medical consumables expenditure	Nursing care expenditure	Bed expenditure	Surgery expenditure	Blood expenditure
Grade II Level A hospitals	− 0.2449*** (128.88)	0.2304*** (71.07)	0.4641*** (148.21)	0.2006*** (34.03)	− 0.0012 (− 12.46)	0.0044** (2.20)	− 0.2333*** (− 15.25)	0.1525*** (5.85)
Reimbursement ratio	2.8096*** (466.71)	3.4378*** (333.59)	2.7881*** (279.88)	2.2562*** (120.87)	1.1782*** (179.22)	1.3802*** (214.34)	1.9099*** (36.60)	0.5015*** (8.40)
Deductible	− 0.0005*** (− 59.34)	− 0.0011*** (− 87.26)	0.0004*** (27.96)	− 0.0018*** (− 77.04)	− 0.0006*** (− 76.16)	− 0.0002*** (− 29.92)	− 0.0007*** (− 10.71)	− 0.0009*** (− 10.31)
Gender	− 0.0233*** (− 17.08)	− 0.0589*** (− 25.25)	0.0084*** (3.81)	− 0.0895*** (− 21.29)	0.0047*** (3.18)	0.0082*** (5.63)	0.1272*** (11.82)	− 0.0782*** (− 5.92)
Age	0.0034*** (105.28)	0.0044*** (79.39)	0.0105*** (199.13)	0.0008*** (7.86)	− 0.0008*** (− 24.23)	− 0.0013*** (− 37.35)	− 0.0011*** (− 4.03)	− 0.0018*** (− 4.79)
Length of stay	0.0374*** (385.04)	0.0440*** (265.43)	0.0142*** (89.60)	0.0187*** (62.46)	0.0565*** (527.33)	0.0581*** (557.89)	0.0131*** (18.62)	0.0008 (1.53)
Disease type	− 0.0098*** (− 6.55)	− 0.0658*** (− 25.79)	− 0.1772 (− 72.64)	0.2493*** (54.11)	− 0.0082*** (− 5.02)	− 0.0159*** (− 10.03)	1.0798*** (55.23)	0.0725*** (3.84)
Time	Controlled	Controlled	Controlled	Controlled	Controlled	Controlled	Controlled	Controlled
Constant term	− 6.0303*** (− 17.87)	− 8.8004*** (− 15.31)	− 1.7797*** (− 3.24)	− 6.3431*** (− 6.13)	3.2551*** (6.29)	3.3614*** (6.66)	− 6.7069*** (− 3.55)	6.9162*** (11.07)
R^2^	0.6328	0.4548	0.4419	0.0867	0.5032	0.5333	0.0460	0.0273
Sample size	488,623	486,558	483,777	483,797	488,166	486,079	117,194	8885

***Significant at 1%,**Significant at 5%, *Significant at 10%, t-statistics in parentheses. The medical expenditure in the model are all calculated on a logarithmic basis

**Table 4:** The effect of Grade II Level B hospitals on medical expenditure

Variables	Medical expenditure							
	Total medical expenditure	Drug expenditure	Diagnostic testing expenditure	Medical consumables expenditure	Nursing care expenditure	Bed expenditure	Surgery expenditure	Blood expenditure
Grade II Level B hospitals	− 0.1113*** ( − 52.70)	− 0.1641*** (− 46.06)	− 0.2481*** (− 71.17)	0.1652*** (25.73)	0.0433*** (19.14)	0.0499*** (22.55)	0.2080*** (13.16)	− 0.1659*** (− 6.09)
Reimbursement ratio	2.8474*** (476.37)	3.4487*** (332.11)	2.8404*** (279.22)	2.3958*** (127.73)	1.1961*** (181.17)	1.4029*** (216.97)	1.9208*** (36.73)	0.4995*** (8.37)
Deductible	− 0.0003*** (− 44.68)	− 0.0010*** (− 80.27)	0.0005*** (42.90)	− 0.0017*** (− 72.48)	− 0.0006*** (− 76.17)	− 0.0002*** (− 26.08)	− 0.0007*** (− 11.39)	− 0.0008*** (− 10.21)
Gender	− 0.0262*** (− 18.97)	− 0.0611*** (− 26.16)	0.0004 (0.18)	− 0.0939*** (− 22.33)	0.0044*** (2.96)	0.0077*** (5.32)	0.1289*** (11.98)	− 0.0789*** (− 5.97)
Age	0.0032*** (97.46)	0.0042*** (76.92)	0.0101*** (188.72)	0.0003*** (3.06)	− 0.0009*** (− 25.71)	− 0.0014*** (− 39.30)	− 0.0011*** (− 4.00)	− 0.0018*** (− 4.80)
Length of stay	0.0374*** (379.57)	0.0439*** (264.10)	0.0141*** (87.46)	0.0191*** (63.52)	0.0566*** (527.87)	0.0582*** (558.64)	0.01293*** (18.42)	0.0008 (1.56)
Disease type	− 0.0113*** (− 7.45)	− 0.0669*** (− 26.15)	− 0.1799*** (− 72.50)	0.2468*** (53.53)	− 0.0084*** (− 5.15)	− 0.0163*** (− 10.22)	1.0813*** (55.29)	0.0722*** (3.83)
Time	Controlled	Controlled	Controlled	Controlled	Controlled	Controlled	Controlled	Controlled
Constant term	− 5.9306*** (− 17.34)	− 8.6132*** (− 14.94)	− 1.5122*** (− 2.71)	− 6.6722*** (− 6.44)	3.2075*** (6.20)	3.3059*** (6.55)	− 6.8434*** (− 3.62)	7.0690*** (11.33)
R^2^	0.6225	0.4515	0.4226	0.0858	0.5036	0.5338	0.0455	0.0276
Sample size	488,623	486,558	483,777	483,797	488,166	486,079	117,194	8885

***Significant at 1%,**Significant at 5%, *Significant at 10%, t-statistics in parentheses. The medical expenditure in the model are all calculated on a logarithmic basis

**Table 5:** The effect of Grade II Level C hospitals on medical expenditure

Variables	Medical expenditure							
	Total medical expenditure	Drug expenditure	Diagnostic testing expenditure	Medical consumables expenditure	Nursing care expenditure	Bed expenditure	Surgery expenditure	Blood expenditure
Grade II Level C hospitals and below	− 0.3531*** (− 80.67)	− 0.1770*** (− 23.86)	− 0.6669*** (− 91.70)	− 1.5519*** (− 71.23)	0.1052*** (22.31)	0.0275*** (5.97)	0.3657*** (6.33)	0.1134 (1.03)
Reimbursement ratio	2.8337*** (476.94)	3.5130*** (341.30)	2.9196*** (290.56)	2.2779*** (124.16)	1.1810*** (180.76)	1.3726*** (215.96)	1.8651*** (35.77)	0.5233*** (8.77)
Deductible	− 0.0004*** (− 48.85)	− 0.0010*** (− 80.18)	0.0004*** (38.96)	− 0.0019*** (− 82.03)	− 0.0006*** (− 75.04)	− 0.0002*** (− 26.35)	− 0.0007*** (− 11.93)	− 0.0008*** (− 10.09)
Gender	− 0.0253*** (− 18.35)	− 0.0615*** (− 26.28)	0.0019 (0.86)	− 0.0846*** (− 20.35)	0.0042*** (2.83)	0.0079*** (5.48)	0.1289*** (11.98)	− 0.0795*** (− 6.00)
Age	0.0032*** (97.67)	0.0041*** (74.45)	0.0106*** (202.17)	0.0009*** (9.76)	− 0.0009*** (− 25.30)	− 0.0013*** (− 37.89)	− 0.0008*** (− 2.76)	− 0.0018*** (− 4.86)
Length of stay	0.0381*** (387.80)	0.0445*** (265.95)	0.0157*** (97.08)	0.0211*** (70.77)	0.0563*** (523.95)	0.0581*** (555.58)	0.0123*** (17.58)	0.0008 (1.45)
Disease type	− 0.1082*** (− 7.16)	− 0.0672*** (− 26.24)	− 0.1792*** (− 72.47)	0.2516*** (55.24)	− 0.0084*** (− 5.21)	− 0.0161*** (− 10.11)	1.0873*** (55.58)	0.0715*** (3.78)
Time	Controlled	Controlled	Controlled	Controlled	Controlled	Controlled	Controlled	Controlled
Constant term	− 6.3151*** (− 18.53)	− 8.9735*** (− 15.54)	− 2.3033*** (− 4.14)	− 6.0105*** (− 6.95)	3.2574*** (6.34)	3.3615*** (6.66)	− 6.2437*** (− 3.31)	7.0561*** (11.28)
R^2^	0.6254	0.4498	0.4265	0.1073	0.5037	0.5334	0.0444	0.0237
Sample size	488,623	486,558	483,777	483,797	488,166	486,079	117,194	8885

***Significant at 1%,**Significant at 5%, *Significant at 10%, t-statistics in parentheses. The medical expenditure in the model are all calculated on a logarithmic basis

**Table 6:** The effect of interaction items on medical expenditure

Variables	Medical expenditure							
	Total medical expenditure	Drug expenditure	Diagnostic testing expenditure	Medical consumables expenditure	Nursing care expenditure	Bed expenditure	Surgery expenditure	Blood expenditure
Hospital level	− 0.2893*** (− 28.42)	0.0392** (2.24)	− 0.2376*** (− 13.89)	0.4808*** (14.87)	0.0938*** (8.39)	− 0.1386*** (− 12.69)	− 1.3494*** (− 9.16)	− 0.2736 (− 1.32)
Interaction item between hospital level and disease type	0.0314*** (10.10)	0.0141*** (2.64)	0.0561*** (11.00)	− 0.0655*** (− 6.62)	− 0.0159*** (− 4.63)	− 0.0302*** (− 9.06)	0.0259 (0.45)	0.1052 (1.39)
Interaction item between hospital level and reimbursement ratio	− 0.0008 (− 0.09)	− 0.3152*** (− 19.34)	− 0.3593*** (− 22.28)	− 0.8498*** (− 28.11)	− 0.1039*** (− 9.99)	0.2058*** (20.25)	1.8337*** (17.42)	− 0.0563 (− 0.32)
Reimbursement ratio	2.8309*** (440.73)	3.5476*** (319.95)	2.9152*** (276.05)	2.4498*** (122.53)	1.1996*** (169.50)	1.3211*** (190.63)	1.5306*** (27.33)	0.5100*** (8.26)
Deductible	− 0.0005*** (− 69.85)	− 0.0012*** (− 92.15)	0.0002*** (15.39)	− 0.0021*** (− 86.76)	− 0.0007*** (− 78.44)	− 0.0002*** (− 27.55)	− 0.0006*** (− 8.92)	− 0.0009*** (− 10.33)
Gender	− 0.0217*** (− 16.08)	− 0.0581*** (− 24.99)	0.0082*** (3.71)	− 0.0854*** (− 20.42)	0.0052*** (3.47)	0.0088*** (6.10)	0.1265*** (11.77)	− 0.0785*** (− 5.93)
Age	0.0034*** (107.14)	0.0043*** (78.89)	0.0106*** (201.84)	0.0009*** (9.61)	− 0.0008*** (− 23.45)	− 0.0012*** (− 36.05)	− 0.0010*** (− 3.65)	− 0.0018*** (− 4.83)
Length of stay	0.0376*** (390.34)	0.0442*** (267.03)	0.0157*** (93.52)	0.0188*** (62.92)	0.0565*** (527.48)	0.0580*** (557.69)	0.0136*** (19.36)	0.0008 (1.49)
Disease type	− 0.0148*** (− 9.36)	− 0.0672*** (− 24.62)	− 0.1853*** (− 71.68)	0.2650*** (54.04)	− 0.0048*** (− 2.73)	− 0.0108*** (− 6.31)	1.0692*** (52.30)	0.0666*** (3.44)
Time	Controlled	Controlled	Controlled	Controlled	Controlled	Controlled	Controlled	Controlled
Constant term	− 5.8602*** (− 17.53)	− 8.7812*** (− 15.29)	− 1.6141*** (− 2.97)	− 6.2706*** (− 6.09)	3.2270*** (6.24)	3.4783*** (6.90)	− 7.0911*** (− 3.76)	7.0770*** (11.33)
R^2^	0.6395	0.4561	0.4533	0.0956	0.5035	0.5342	0.0485	0.0267
Sample size	488,623	486,558	483,777	483,797	488,166	486,079	117,194	8885

***Significant at 1%,**Significant at 5%, *Significant at 10%, t-statistics in parentheses. The medical expenditure in the model are all calculated on a logarithmic basis

The empirical results of multivariate regression analysis are shown in Table 6. Per the empirical results, after the addition of interaction items, the effect of the hospital level on total medical expenditure, diagnostic testing, bed and surgery expenditure were significantly negative (*p* < 0.001). However, the effect of the hospital level on drug, medical consumables and nursing care expenditure were significantly positive (*p* < 0.001). The effect of the interaction item between hospital level and disease type on total medical expenditure was significantly positive (*p* < 0.001), while the effect on medical consumables, nursing care and bed expenditure were significantly negative (*p* < 0.001). Due to the higher cost of high-level hospitals, the more serious the disease was, the higher the total medical expenditures were. The effect of the interaction item between hospital level and reimbursement ratio on total medical expenditure was significantly negative (*p* < 0.001), while the effect of the interaction item between hospital level and reimbursement ratio on bed and surgery expenditure was significantly negative (*p* < 0.001). The results indicated that the higher the hospital level was, and the higher the reimbursement ratio was, the lower the total medical expenditures of drug expenditure, diagnostic test expenditure, medical consumable expenditure and nursing expenditure were. The effect of the reimbursement ratio, deductible, gender, age, length of stay and disease type on medical expenditure were consistent with the previous analysis.

### Robustness check

In order to test the robustness of the regression results, the dynamic generalized moment estimation method (GMM) was used for parameter estimation. The regression results are shown in Table 7. The regression results were consistent with the previous regression results. Among them, the signs and significance levels of the explanatory variables such as hospital level, reimbursement ratio, the interaction between hospital levels and disease type, and the interaction between hospital levels and the reimbursement ratio were consistent with the previous regression results.

**Table 7:** GMM empirical results of hospital level on medical expenditure

Variables	Medical expenditure							
	Total medical expenditure	Drug expenditure	Diagnostic testing expenditure	Medical consumables expenditure	Nursing care expenditure	Bed expenditure	Surgery expenditure	Blood expenditure
Hospital level	− 0.2665*** (− 27.21)	0.0029 (0.18)	− 0.1737*** (− 8.83)	0.6074*** (17.06)	0.2073*** (16.59)	− 0.1468*** (− 14.01)	− 1.3639*** (− 9.57)	− 0.2807 (− 1.45)
Interaction item between hospital level and disease type	0.0395*** (10.16)	0.0154*** (3.56)	0.0544*** (10.32)	− 0.0687*** (− 6.73)	− 0.0187*** (− 5.12)	− 0.0297*** (− 10.15)	0.0252 (0.43)	0.1063 (1.41)
Interaction item between hospital level and reimbursement ratio	− 0.0456*** (− 4.70)	− 0.2195*** (− 12.91)	− 0.4968*** (− 25.75)	− 1.0707*** (− 29.65)	− 0.3166*** (− 25.44)	0.2391*** (21.05)	1.8055*** (19.04)	− 0.0554 (− 0.36)
Reimbursement ratio	2.8639*** (104.50)	3.4897*** (97.41)	3.0035*** (181.38)	2.5526*** (102.23)	1.3029*** (38.28)	1.2942*** (32.73)	1.6225*** (27.47)	0.5205*** (8.44)
Deductible	− 0.0005*** (− 63.59)	− 0.0012*** (− 83.60)	0.0002*** (14.07)	− 0.0021*** (− 84.77)	− 0.0007*** (− 67.91)	− 0.0002*** (− 22.20)	− 0.0006*** (− 9.30)	− 0.0009*** (− 10.12)
Gender	− 0.0222*** (− 16.32)	− 0.0572*** (− 24.42)	0.0071*** (3.16)	− 0.0854*** (− 20.42)	0.0026* (1.67)	0.0090*** (6.13)	0.1256*** (11.67)	− 0.0774*** (− 5.87)
Age	0.0034*** (94.57)	0.0043*** (70.72)	0.0105*** (167.93)	0.0009*** (9.61)	− 0.0009*** (− 18.49)	− 0.0012*** (− 29.03)	− 0.0009*** (− 2.66)	− 0.0018*** (− 4.70)
Length of stay	0.0369*** (26.52)	0.0445*** (25.07)	0.0136*** (24.15)	0.0175*** (21.57)	0.0554*** (32.12)	0.0582*** (28.66)	0.0123*** (10.79)	0.0007 (1.18)
Disease type	− 0.0133*** (− 10.22)	− 0.0727*** (− 32.26)	− 0.1792*** (− 89.13)	0.2705*** (61.89)	0.0016 (0.96)	− 0.0134*** (− 8.52)	1.0783*** (54.24)	0.0662*** (3.58)
Constant term	5.5159*** (488.71)	3.9204***(246.49)	3.7763***(339.61)	2.6889*** (148.19)	3.4860*** (255.49)	3.7007*** (240.35)	2.4218*** (38.25)	7.0801*** (103.57)
R^2^	0.6368	0.4491	0.4434	0.0863	0.4681	0.5304	0.0466	0.0262
Sample size	488,623	486,558	483,777	483,797	488,166	486,079	117,194	8885

***Significant at 1%,**Significant at 5%, *Significant at 10%, t− statistics in parentheses. The medical expenditure in the model are all calculated on a logarithmic basis

## Discussion

From the perspective of the COH framework, combining hospital characteristic factors, price factors and patients characteristic factors, and using UEBMI data of Chengdu City from 2011 to 2015, our study empirically analyzed the effect of COH on medical expenditures by multivariate regression modeling. This study had a number of important findings.

Firstly, the hospital level had a significant influence on medical expenditure. The higher the hospital level, the lower the medical expenditures. This could be explained based on the following reasons: (a) The government has more strict supervision over the high-level hospitals than the low-level hospitals, and the medical price of high-level hospitals was relatively reasonable, (i.e. more rreflective of the true cost). This translates into a lower likelihood of excessive medical treatment and less unreasonable medical expenditure (overuse drugs and diagnostic testing.); (b) High- level hospitals were more standardized than low-level hospitals in terms of management systems and treatment processes; (c) High-level hospitals received more government subsidies than low-level hospitals. In order to generate profit, low-level hospitals prescribed more drugs and provided more high-technology tests. There may also have been over-prescribing behavior of physicians at low-level hospitals; (d) In China, there are more medical experts and more advanced medical equipment in high-level hospitals, so it is generally believed that healthcare service quality of the high-level hospitals were better. The provision of medical services was more efficient in high-level hospitals, saving on medical expenditures. The higher surgery expenditures may be related to the rigid price system for different COH in China.

Secondly, the reimbursement ratio of UEBMI had a significantly positive effect on various types of medical expenditures, indicating that the higher the reimbursement ratio was, the higher the medical expenditure would be. Therefore, the government should set the reimbursement ratio of medical insurance within a rational range which will enhance financial risk protection and improve population health, This in turn would not only control the growth of unreasonable medical expenditure, but also incentivize hospitals in getting more profit from medical insurance. The effect of the deductible on various types of medical expenditures was significantly negative, indicating that the higher the deductible was, the lower the medical expenditure. One possible explanation was that the higher deductible, the higher the share of out of pocket (OOP) expenditure. This may generate negative incentives for patients when seeking necessary medical services.

Thirdly, the effect of gender and age on medical expenditures was inconsistent. On the one hand, the older the age was, the higher the medical expenditure of drug, diagnostic testing and medical consumables. Conversely the older the age, the lower the nursing care, bed, surgery and blood expenditure. This may be related to the large proportion of elderly in the sample in this paper. Some studies have demonstrated that the time-to-death is a significant factor in medical expenditures [40, 41, 46]. The length of stay had a significantly positive effect on medical expenditure. The more serious the disease type, the higher the medical expenditures of, drug, diagnostic testing, nursing care and bed expenditure. Interestingly, the more serious the disease type, the lower the medical consumables, surgery and blood expenditures. This may be related to the large proportion of elderly in the sample used. Influenced by traditional Chinese culture, the elderly with more serious diseases (e.g. terminal cancer) may forego treatment.

Finally, after distinguishing for COH, hospitals of different levels had different influences on medical expenditures. In general, the higher the level of hospitals, the lower the total medical expenditure. After the addition of interaction items,including hospital levels and disease type and; hospital levels and the reimbursement ratio, the effect of hospital level, disease type and reimbursement ratio on medical expenditures was inconsistent. The higher the hospital level along with disease severity, and the higher the medical expenditure of, drugs and diagnostic testing. The higher the hospital level was, along with a high the reimbursement ratio, the lower the medical expenditures of drug, diagnostic testing, medical consumables and nursing care. This may be due to the relatively standardized hospital management system and treatment processes, which would save on medical expenditures. Thus China should strengthen hospital management (strict supervision and information technology) and clarify the standards of different classfications of hospitals to ensure that paitents can get effective treatment.

In general, the larger the scale of the hospital, the more resources they had at their disposal, including advanced medical devices, and management systems. At the same time, these types of hospitals received more government subsidies and medical insurance funding; as well as benefitted from more formalized and structured operational systems. Lower-level hospitals had less government subsidies, operated fewer hospital beds and had less advanced technology. In order to generate profit, lower-level hospitals overly prescribed drugs and high-technology tests. Therefore, the medical expenditures of the high-level hospital was not always higher than that of the low-level hospital. Thus per the severity of the disease, different diseases should be treated in different levels of hospitals.

Our findings are consistent with other studies. Newhouse [4] studied the factors driving increased medical expenditure. He found that factors affecting medical expenditures can be roughly divided into three levels. The first level mainly referred to price factors, such as the medical insurance system [5–13], government subsidies [14–17], essential medicines programmed (EMP) [18–20] and separation of hospital revenue from drug sales [21–23]. These policies influence medical expenditures by affecting the relative or absolute prices of medical services and product [24]. The second level mainly referred to hospital characteristic factors, such as the diagnosis and treatment level [25–27], hospital management [28–33] and use of advanced medical devices [34–39]. The third level mainly referred to the patients characteristics, such as age [40], gender [41], education status [42], and health status [43–45]. In summary, our study found price, hospital characteristic and patients factors affected medical expenditures, consistent with Newhouse.

Our study has some interesting findings which can potentially be used for policy recommendations. As it relates to hospital management in China, the hospital level has become an important factor influencing the effective allocation of health resource. In order to improve the quality of medical services and control the increase of medical expenditure, the classification of hospital should become an important focus of health-care reform. Secondly, China should focus on improvements to the hospital management system and medical insurance system, and strengthen the government oversight of hospitals of different levels. In addition, the medical insurance system should be designed to encourage behaviors of physicians and hospitals in order to improve the quality and efficiency of medical services. Further, China examines the marginal effect of factors influencing medical expenditures in order to adopt reform of the healthcare system. Lastly China should implement differentiated reimbursement ratios for different disease types treated in different hospitals levels in order to improve medical insurance reimbursement policies.

Our study has several important contributions. Firstly, the use of the UEBMI data of Chengdu, is the first time a study on the effect of COH on medical expenditure has been undertaken. Secondly, establishing a framework of COH, which can be divided into hospital characteristic factors, price factors and patients characteristic factors was unique. Finally, by adopting multivariate statistical analysis and introducing interaction items into the model, we could better describe the mechanism of the effect of hospital grade, reimbursement ratio, and disease type on medical expenditures. This can be of use for the implementation and improvement of a tiered delivery system. However, the effect of Grade II Level A hospitals, Grade II Level B hospitals, Grade II Level C hospitals and below on different types of medical expenditures were different, needs to be further analyzed.

Our study also has some limitations. First, due to the data limitations, we only studied Chengdu City and the patients were mainly elderly, which may overestimate the effect of COH on HE. Second, the current HE don’t include ER visit and medications. Third, also due to the data limitations, we didn’t address Grade I and III hospitals, only Grade II Level A, B and C hospitals. This in turn may underestimate the effect of COH on HE. Although we only used data with Grade II Level A, B and C hospitals, we could still have similar findings with Grade I and III hospitals. On the one hand, different grade hospitals had the same classification standards and management system in China, such as hospital’s scale, service provision, medical technology and equipment, medical research and so on. The differences among different grade hospitals were very similar. On the other hand, there were many different grade hospitals with a large number of patients in China. According to China's Health Statistics Bulletin in 2019 [47], we found that the number of Grade I, II and III hospitals were 11,264, 9,687 and 2,749 and the number of patients were 12,090,000, 81,770,000 and 92,920,000, respectively. The number of Grade II hospitals were large enough to stand for Grade I and III hospitals to estimate the effect of COH on HE. While there are differences, the effect of COH on HE is an important topic in China, and any evaluation provides important insights. Finally, our data was a compilation of non-severe to severe DRGs (within the same DRG) and associated costs by severity could not be examined.

## Conclusion

In summary, this study measured the impact of different COH on medical expenditures using UEBMI data of Chengdu City from 2011 to 2015. Our study empirically analyzed the effect of COH on medical expenditures using multivariate regression analysis. Our findings may contribute to the body of the knowledge on the impact of COH on medical expenditure in China. Hospitals of different level have different influences on medical expenditure. In the process of attempting to reduce medical expenditures, the government address the medical insurance payment. Meanwhile, the government should also focus on improving the health of residents and in directing patients to appropriate levels of care.

## Figures and Tables

**Fig. 1:**
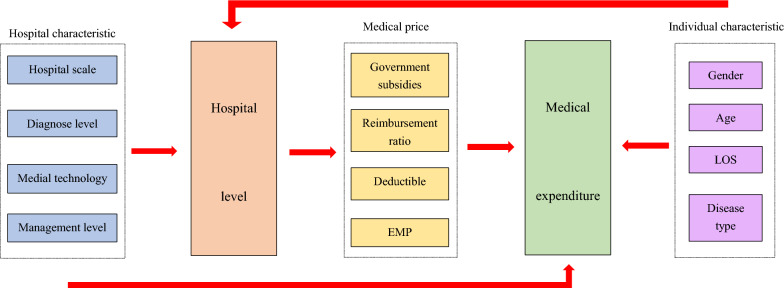
A classification of hospital framework: hospital characteristic, price and individual characteristic

**Fig. 2:**
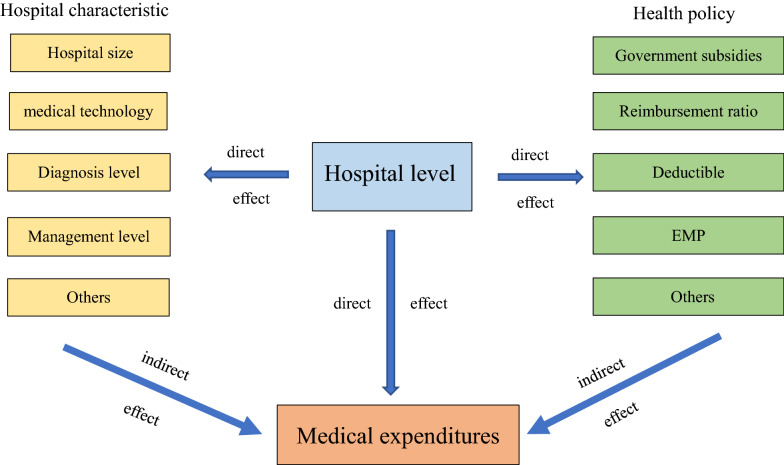
The mechanism of effect of hospital level on medical expenditures

## Data Availability

Data and materials are from Healthcare Security Administration of Chengdu City.

## Appendices

### Appendix 1

See Fig 1.

### Appendix 2

See Fig 2.

## Abbreviations

UEBMI: Urban Employee Basic Medical Insurance
COH: Classification of Hospitals;
GDP: Gross Domestic Product
ZMDP: Zero-markup drug policy
